# Accuracy of Visual Tests for Primary Cervical Cancer Screening in Rural Nepal

**DOI:** 10.31729/jnma.3857

**Published:** 2018-12-31

**Authors:** Niresh Thapa, Muna Maharjan, Girishma Shrestha, Narayani Maharjan, Deborah Lindell, Na Zuo, Jing Yang, Ninu Maskey, Hongbing Cai

**Affiliations:** 1 Department of Gynecological Oncology, Zhongnan Hospital of Wuhan University, Hubei Cancer Clinical Study Center, Hubei Key Laboratory of Tumor Biological Behaviors, Wuhan, P. R. China; 2 Karnali Academy of Health Sciences, Jumla, Nepal; 3 HOPE School of Nursing, Zhongnan Hospital of Wuhan University, Wuhan, Hubei, China; 4 Patan Hospital, Patan Academy of Health Sciences, Nepal; 5 Department of Clinical Laboratory Science, Zhongnan Hospital of Wuhan University, Wuhan, Hubei, China; 6 Frances Payne Bolton School of Nursing, Case Western Reserve University, Cleveland, Ohio, USA; 7 Department of Haemato-Pathology, Zhongnan Hospital of Wuhan University, Wuhan, China

**Keywords:** *accuracy*, *cervical cancer*, *cytology*, *screening*, *visual tests*

## Abstract

**Introduction:**

In Nepal, cervical cancer is the most common female cancer. Unfortunately, there is no uniform effective screening system available all around the country. The objective of this study is to evaluate the cytology, Visual Inspection with Acetic Acid and with Lugol's Iodine alone or in combination to detect a pre-cancerous lesion in rural Nepal.

**Methods:**

It is an analytical cross-sectional study. Convenience sampling technique was used to select participants who were apparently healthy, married, non- pregnant women of aged 20–65 years for cervical cancer screening program. Screening tests were performed on all eligible women (n=2143) after socio-demographic and reproductive health data collection. A biopsy was applied as a gold standard test. Cross-tabulations were used to describe the test sensitivity, specificity, positive predictive value, and negative predictive value at a 95% confidence interval. Diagnostic odds ratio was also calculated.

**Results:**

A majority, 2143 (94%), of women accepted and participated in this study. The sensitivity vs specificity of cytology, VIA, and VILI was 57.1% vs 98.3%, 71.4% vs 88.8% and 78.6% vs 85.1%, and of the co-testing of ‘Both positive VIA and VILI’ and ‘Either positive VIA or VILI’ was 64.3% vs 85.7% and 90.1% vs 83.7% respectively. Negative predictive value of all tests exceeded 99.7%. Cytology had the highest Diagnostic odds ratio (64.9), followed by the co-test ‘Either positive VIA or VILI’ (27.7).

**Conclusions:**

Cervical cancer screening by co-testing ‘Either positive VIA or VILI’ is more useful than cytology; VIA and or VILI are easy, safe, feasible and well-accepted tests in a low resource setting, Nepal.

## INTRODUCTION

Cervical cancer is the fourth most common female cancer among women in the world with yearly incidence and mortality of 527,624 and 265,672 respectively.^1^ In Nepal, cervical cancer is the most common female cancer.^1,2^ Unfortunately, there is no uniform effective screening system easily available all around in the country.

Reports indicate only 5% of Nepalese women have ever had a cervical smear test and the proportion of unscreened women is much higher among women who are illiterate and live in rural regions,^3^ in spite of Visual Inspection with Acetic Acid (VIA) being the screening test in national guideline only 0.9% of women recognized VIA as a cervical cancer screening method.^4,5^

Data on population-based comparative study regarding the accuracy, acceptability, and feasibility of visual tests are scarce in Nepal. So the objective of this study is to evaluate the cytology, Visual Inspection with Acetic Acid (VIA) and with Lugol's Iodine (VILI) alone or in combination to detect a pre-cancerous lesion in rural Nepal.

## METHODS

This analytical cross-sectional study was conducted in Jumla, a remote, mountainous district of Karnali state in Nepal from May 2016 to January 2017.

There are seven rural municipalities and one municipality in Jumla district. The total population is 108921, with the female population of 54023. The approximate eligible female population is 21400^6^ excluding pregnant and unmarried women. The sample size was calculated as 10% of the eligible population that is 2100. Convenience sampling technique was used to select the study participants.

This study was approved by the ethical review board of Nepal Health Research Council, Nepal. Population-based opportunistic cervical cancer screening was conducted. The detailed methodology including flow diagram was explained in our previous study.^7^

An educational program was conducted to raise awareness of cervical cancer and encourage participation in the screenings. Written informed consent was obtained from the respondents who were willing to participate. A structured questionnaire was used to obtain socio-demographic and reproductive health information. Inclusion criteria included women of aged 20–65 years, apparently healthy, married and with no past history of cervical cancer. Exclusion criteria included pregnant or symptomatic women seeking any kind of gynecologic health care and those who wished to withdraw from the study.

The Bethesda system 2001 was used to report cytology results.^8^ Low-grade squamous intraepithelial lesion (LSIL) or worse was considered to be abnormal or positive. VIA and VILI were considered to be positive according to the criteria (Table 1).^9^ World Health Organization (WHO) Classification of Tumors of Female Reproductive Organs, 2014 was used to report the biopsy result.^10^

Histology was regarded as the gold standard for defining final disease status. True positive (TP) was defined as positive by both histology and screening test; true negative (TN) was defined as negative by both histology and screening test; false positive (FP) was defined as negative by histology but positive by screening test; and false negative (FN) was defined as positive by histology but negative by screening test. Quality control measures were taken in all levels of the study. The pathologist was blinded with cytology and VIA/VILI reports.

The disease was managed based on biopsy or cytology report (for participants who did not have a biopsy report). Women with invasive cancers were referred to a cancer center for further management. Women with high-grade squamous intraepithelial lesion (HSIL) were recommended for treatment with cryotherapy or loop electrosurgical excisional procedure (LEEP) or cone excision, or hysterectomy. Women with LSIL were given a choice of immediate treatment or follow-up after 6 months.

Data analysis was performed using the Statistical Package for the Social Sciences (SPSS version 20.0; IBM Corp., Armonk, NY, USA). Cross-tabulations were used to describe the test sensitivity, specificity, positive predictive value (PPV), and negative predictive value (NPV) at 95% confidence interval (CI). Combinations of two visual tests were calculated for sensitivity, specificity, PPV, and NPV.

**Table 1:** VIA and VILI Criteria for Positive and Negative.

Test	Positive	Negative
VIA^*^	One minute after application of acetic acid: Distinct, well-defined, dense, acetowhite lesions close to the SCJ† or the external os if SCJ was not visible Dense acetowhite lesion in the columnar epithelium or entire cervix Pre-existing condyloma or leukoplakia turning intensely white Ulceroproliferative growth turning acetowhite	One minute after application of acetic acid: No distinct acetowhite lesions, ill-defined or faint patchy, bluish-white, shiny, pinkish-white or doubtful lesions Dot or streak-like acetowhitening on the columnar epithelium Cervical polyps or Nabothian cysts appeared as bluish-white acetowhite or button like lesion Satellite lesions- distant from SCJ
VILI^‡^	After the application of iodine solution Well-defined dense, thick, bright, mustard- or saffron-yellow, iodine non-uptake areas seen in the transformation zone, close to the SCJ. Entire cervix turned densely yellow Ulceroproliferative growth turning densely yellow.	After the application of iodine solution A normal cervix, the squamous epithelium turned mahogany brown or black and no change in the columnar epithelium Patchy, indistinct, ill-defined colorless or partially brown areas in SCJ. Pale areas of no or partial-iodine uptake areas on polyps Satellite, thin, yellow, non-iodine uptake areas with angular, or digitating margins, resembling geographical areas seen distant from the SCJ.

* Visual Inspection with Acetic Acid;

† Squamo-columnar junction;

‡ Visual Inspection with Lugol's Iodine

Two conditions were analyzed: first is “both test positive”, a positive result means having both tests positive and a negative result means negative in either one; second is “either test positive”, a positive result means positive in at least one of the test and a negative result means negative in both tests. Diagnostic odds ratio (DOR) was calculated using the formula, DOR = sensitivityxspecificity/(1-sensitivity) (1-specificity).^11^

## RESULTS

The total of 2279 women participated in counseling session among them 2143 (94%) agreed to participate in this study. The participants' median age and median marital age was 30 years and 16 years respectively. Forty-three percent of women had more than or equal to four pregnancies. Illiterate and women with informal education occupied 1577 (73.6%), and only 566 (26.4%) had formal education (Table 2).

**Table 2:** Characteristics of the participants (n = 2143).

Characteristics	n (%)
**Age (year)**	
20–34	1330 (62.1)
35–49	665 (31.1)
50–65	148 (6.9)
Median age	30
**Marital age (year)**	
≤ 19	1768 (82.5)
≥ 20	375 (17.5)
Median marital age	16
**Number of pregnancy**	
0–3	1221 (57)
≥ 4	922 (43)
**Education**	
Illiterate / Informal education	1577 (73.6)
Formal education	566 (26.4)

Table 3 demonstrates the positive rate of screening tests. Around 2113 (96%) participants were eligible for further analysis of all test reports- VIA, VILI, and Cytology. The positive rates of cytology, VIA, and VILI among women aged 20–34 years were 34 (2.4%), 199 (14.8%) and 263 (19.6%) respectively. Likewise, the positive rates of cytology, VIA, and VILI among women aged 50–65 years were 10 (8.8%), 6 (4.4%) and 12 (8.8%) respectively. The positive rate of VIA and VILI tended to decrease with increase in age whereas cytology showed the opposite trend.

**Table 3:** Positive rates of screening tests according to the age groups.

Characteristics	Number n (%)	Cytology (n§ = 2113) n (%)	VIA (n = 2143) n (%)	VILI (n=2143) n (%)
Age (years)				
20–34	1341 (62.6)	34 (2.4)	199 (14.8)	263 (19.6)
35–49	665 (31)	34 (5.0)	62 (9.3)	87 (13.1)
50–65	137 (6.4)	10 (8.8)	6 (4.4)	12 (8.8)

§ *Out of 2143 samples, 30 samples were not adequate for analysis in cytology test.*

Table 4 presents the final disease status by gold standard test compared with screening tests. A biopsy was taken for a positive result of VIA or VILI or both. Total around 63 (17%) (out of 371 eligible biopsy result) biopsy result was positive. Out of 62 positive biopsy results: LSIL was 41 (11.1%), HSIL was 14 (3.8%) and invasive cancer was 7 (1.9%).

Table 5 shows the accuracy of a screening test to detect HSIL. The sensitivity of cytology, VIA, and VILI was 57.1%, 71.4%, and N 78.6% respectively. The specificity of cytology, VIA, and VILI was 98.3%, 88.8%, and 85.1% respectively. Positive predictive value (PPV) of cytology, VIA, and VILI was 18.6%, 4.1%, and 3.4% respectively. Negative predictive value (NPV) of all three tests exceeded 99.7%. The co-testing of visual tests ‘both positive VIA and VILI’ had 64.3% sensitivity, 90.1% specificity, and 4.2% PPV while ‘Either positive VIA or VILI’ had 85.7% sensitivity, 83.7% specificity, and 3.4% PPV. NPV of both combination tests exceeded 99.7%. Cytology had the highest DOR that is 64.9, followed by the co-test ‘Either positive VIA or VILI’ had DOR 27.7. Co-testing ‘Both positive VIA & VILI’ had the lowest DOR 16.0.

**Table 4:** Final disease status by histology report compared with screening tests.

Test Result	Histology Report (Gold Standard)				Total
	Cancer	HSIL	LSIL	Normal	
**Cytology**					
**Abnormal**	5	8	30	35	78
**Normal**	2	6	11	2016	2035
**Total**	7	14	41	2051	2113
**VIA**					
**Positive**	4	10	20	233	267
**Negative**	3	4	21	1848	1876
**Total**	7	14	41	2081	2143
**VILI**					
**Positive**	5	11	35	311	362
**Negative**	2	3	6	1770	1781
**Total**	7	14	41	2081	2143

*HSIL, high-grade squamous intraepithelial lesion; LSIL, low-grade squamous intraepithelial lesion; VIA, Visual Inspection with Acetic Acid; VILI, Visual Inspection with Lugol's Iodine.*

**Table 5:** Accuracy of screening test/tests to detect HSIL.

Test	Sensitivity (%) (95% CI)	Specificity (%) (95% CI)	PPV (%) (95% CI)	NPV (%) (95% CI)	DOR
Cytology	57.1 (28.8–82.3)	98.3 (97.6–98.8)	18.6 (11.5–28.5)	99.7 (99.4–99.8)	64.9
VIA	71.4 (41.9–91.6)	88.8 (87.4–90.1)	4.1 (2.9–5.7)	99.8 (99.5–99.9)	17.9
VILI	78.6 (49.2–95.3)	85.1 (83.4–86.6)	3.4 (2.6–4.5)	99.8 (99.5–99.9)	20.1
Both Positive VIA & VILI	64.3 (35.1–87.2)	90.1 (88.8–91.4)	4.2 (2.8–6.2)	99.7 (99.5–99.8)	16.0
Either Positive VIA or VILI	85.7 (57.2–98.2)	83.7 (82.1–85.3)	3.4 (2.7–4.3)	99.8 (99.6–99.9)	27.7

*HSIL, high grade squamous intraepithelial lesion; VIA, Visual Inspection with Acetic Acid; VILI, Visual Inspection with Lugol's Iodine; PPV, positive predictive value; NPV, Negative predictive value; DOR, Diagnostic Odds Ratio.*

All biopsy positive cases (n = 62) were managed according to the study protocol as mentioned in our previous report.^7^ Among LSIL cases (n=41): 21 patients opted repeat test in six months and the remaining 20 patients received cryotherapy. Among the HSIL cases (n=14): LEEP was done for eight cases; simple hysterectomy for four cases and two patients refused any treatment. No significant complications were observed during screening and treatment. Patients with invasive cervical cancer (n=7) were referred to a cancer center for further management.

## DISCUSSION

The current study compared the accuracy of cytology, VIA and VILI alone or in combination among apparently healthy, asymptomatic and previously unscreened women in a poor resource setting. Our findings showed that visual tests, VIA and VILI, can be a very good alternative for primary cervical cancer screening. Among the visual tests, VILI was superior to VIA. Though the specificity and PPV were lower VILI had higher sensitivity to detect cervical pre-cancer than VIA and cytology. Combination test: ‘Either Positive VIA or VILI’ seemed to have a better balance of sensitivity and specificity. Though slightly less specific (14.6%) than cytology, ‘Either Positive VIA or VILI’ was 26.6% more sensitive. The main advantages of these visual tests (easy, safe, cost-effective, instant report, and no need of advance technology/equipment or human resource) are extremely in favor of low resource settings. Certainly, there are some disadvantages, such as overtreatment or unnecessary anxiety among women who had a false positive report by visual tests due to its lower PPV (3.4%-4.1%) and relatively lower specificity (85.1%-88.8%). Visual tests alone or in combination can be applied at least to rule out the cervical neoplasia, is its high NPV (more than 99.7%).

This study evaluated the accuracy as well as acceptability and feasibility of visual tests as alternative methods for cervical cancer screening in a rural setting. Despite being the most common female cancer in Nepal, the rate of cervical cancer screening is below 5% and the scenario is worse in rural and poor resource settings.^3^ A national cervical cancer screening guideline (VIA method) has been in place since 2010. But there is only one published Nepalese study in 2007 reporting the accuracy of visual tests which was conducted among a limited number of women (n=300) in the gynecologic out-patient clinic of tertiary care center in the capital city.^12^ Most population-based studies were conducted in other South Asian or sub-Saharan countries which are socio-culturally different than Nepal.

Findings of VIA and or VILI in this study are consistent with several studies conducted in India and China^11,13^ but sensitivity and specificity were higher in studies conducted in the sub-Saharan African country.^14^ Nessa et al. from Bangladesh reported that VIA had higher sensitivity (93.6%) but lower specificity (58.3%) than our findings.^15^ The positive rate of VIA and VILI showed a decreasing trend among older women. The lowest positive rate of VIA (4.4%) was observed among the age group of 50–65 years, which is similar to the previous reports.^13,16^ The lower positive rate among older women may be related to the smaller number of participants in that age group or difficult visibility of SCJ in post-menopausal women.

The higher sensitivity of VILI compared to VIA might be due to easier to detect the yellow color changes produced by Lugol's iodine compared to the aceto-whitening observed after the application of acetic acid. There were other advantages of VILI such as no interval between application of Lugol's iodine and appearance of a mustard yellow lesion and no complaint of burning sensation (which was usually observed with acetic acid).

The sensitivity and specificity of the cytology in this study were within the range of other studies.^13,17^ In Norway, where four pathologists at three hospitals, read 100 pap smears, at the threshold for CIN2 +, the sensitivity varied from 68.8% to 93.8% and specificity from 70.6% to 95.6%. It was concluded in that study that cytology based cervical cancer screening has limited accuracy.^18^ Moreover, establishing a nationwide quality-assured cytology-screening program might be very challenging and logistically almost impossible in rural settings of Nepal. HPV DNA test has better sensitivity and specificity so that it can be a good option for screening provided it is easily available and free of cost. Unfortunately, it is not possible at present in Nepal as pointed out in a recent report. Financial and logistics problem are the major issues. HPV DNA test demands high cost, advanced setting, and highly skilled human resource.^4^ This is not in favor of low resource setting and difficult to implement as national policy in low and middle-income countries.

Barriers such as high costs and low public awareness prohibit the introduction of prophylactic vaccines in a country like Nepal. Therefore, effective screening programs are only viable means of rapidly reducing the heavy burden of cervical cancer.^19^ An 18-year follow-up of the Guanacaste cohort women, who had screening with HPV, cytology, and visual methods, showed an additional 31% invasive cervical cancer incidence reduction with apparent down staging of cancers.^20^ Another report from a randomized trial conducted in South India showed VIA to be associated with a 25% reduction in cervical cancer incidence and a 35% reduction in mortality from cervical cancer.^21^ A study of patients with HIV from Kenya reported VIA alone appears to be a more suitable strategy for cervical cancer screening,^16^ while a recent meta-analysis reported VILI alone appeared to be the most useful visual screening strategy.^22^

A limitation of this study was verification bias, as the gold standard test was not applied in all participants.

Detection of true positive ‘HSIL’ by each screening test was used for the calculation of sensitivity, specificity, PPV, and NPV. Therefore, these values could be viewed as approximate estimations. We were not able to use colposcopy to take the biopsy or as reference standard which might have resulted in missing few cases. However, when colposcopy is not available at the primary care level, VIA guided cervical punch biopsy can be used.^23^ Nevertheless, our findings of this study are comparable with other similar studies conducted in low resource settings.

## CONCLUSIONS

Cervical cancer screening by co-testing ‘Either positive VIA or VILI’ is more useful than cytology; VIA and or

VILI are easy, safe, feasible and well-accepted tests in a low resource setting, Nepal. However, careful implementation and standardized periodic training for health personnel are warranted to ensure quality outcomes.
